# Acclimation of photosynthetic apparatus in the mesophilic red alga *Dixoniella giordanoi*

**DOI:** 10.1111/ppl.13489

**Published:** 2021-07-05

**Authors:** Nicolò Fattore, Simone Savio, Antoni M. Vera‐Vives, Mariano Battistuzzi, Isabella Moro, Nicoletta La Rocca, Tomas Morosinotto

**Affiliations:** ^1^ Department of Biology University of Padova Padova Italy; ^2^ Centro di Ateneo di Studi e Attività Spaziali (CISAS) “Giuseppe Colombo” University of Padova Padova Italy

## Abstract

Eukaryotic algae are photosynthetic organisms capable of exploiting sunlight to fix carbon dioxide into biomass with highly variable genetic and metabolic features. Information on algae metabolism from different species is inhomogeneous and, while green algae are, in general, more characterized, information on red algae is relatively scarce despite their relevant position in eukaryotic algae diversity. Within red algae, the best‐known species are extremophiles or multicellular, while information on mesophilic unicellular organisms is still lacunose. Here, we investigate the photosynthetic properties of a recently isolated seawater unicellular mesophilic red alga, *Dixoniella giordanoi*. Upon exposure to different illuminations, *D. giordanoi* shows the ability to acclimate, modulate chlorophyll content, and re‐organize thylakoid membranes. Phycobilisome content is also largely regulated, leading to almost complete disassembly of this antenna system in cells grown under intense illumination. Despite the absence of a light‐induced xanthophyll cycle, cells accumulate zeaxanthin upon prolonged exposure to strong light, likely contributing to photoprotection. *D. giordanoi* cells show the ability to perform cyclic electron transport that is enhanced under strong illumination, likely contributing to the protection of Photosystem I from over‐reduction and enabling cells to survive PSII photoinhibition without negative impact on growth.

## 1 INTRODUCTION

Organisms performing oxygenic photosynthesis exploit sunlight to fix CO_2_ into biomass thanks to the activity of photosystem (PS) I and II, two multiprotein supercomplexes located in the thylakoid membranes. Both photosystems involved in oxygenic photosynthesis are composed of a core complex responsible for the charge separation and electron transfer reactions that are highly conserved among photosynthetic organisms, both prokaryotic and eukaryotic. Photosystems also include an antenna system to increase the light‐harvesting capacity. Antenna proteins diverged during evolution, possibly also due to adaptation to different ecological niches. In cyanobacteria and red algae, the ability to capture light is increased by soluble protein complexes, called phycobilisomes (PBS), that bind pigments like phycocyanin and phycoerythrin. In most eukaryotes, instead, the antenna system is composed of hydrophobic light harvesting complexes (LHC) proteins, localized in the thylakoid membranes and bind chlorophyll (Chl) and carotenoids (Car). In different eukaryotic groups, the LHC themselves diversified into various subfamilies, such as LHCA/B found in green algae and plants or LHCF in diatoms (Büchel, 2015).

Photosynthetic activity is highly influenced by environmental factors like illumination intensity, temperature, CO_2_, and nutrient availability. Many environmental conditions can drive the photosystems to saturation and over‐reduction, with the possible production of various Reactive Oxygen Species (ROS) causing oxidative damage of the photosynthetic apparatus (photoinhibition) (Li et al., 2009; Murata et al., 2007; Szabó et al., 2005). To thrive in highly variable environmental conditions, photosynthetic organisms evolved multiple mechanisms to modulate photosynthetic activity depending on the environmental conditions, limiting over‐reduction and ROS formation. These regulatory mechanisms have different activation timescales, enabling responses to short‐term and long‐term dynamics of the environmental factors (Eberhard et al., 2008; Walters, 2005). The long‐term acclimation response involves modulation of the composition of the photosynthetic apparatus according to irradiance intensity, regulating Chl and Car content (Falkowski & LaRoche, 1991; Walters, 2005). On a shorter timescale, photosynthetic organisms instead activate mechanisms for the modulation of light harvesting efficiency, such as non‐photochemical quenching (NPQ), driving the dissipation of excess excitation energy as heat (Li et al., 2009). Strong illumination also activates the xanthophyll cycle, where zeaxanthin is synthesized from pre‐existing violaxanthin, further contributing to the protection from ROS (Havaux et al., 2007). Photosynthetic electron transport is also regulated by the presence of alternative electron transport mechanisms, such as cyclic electron flow (CEF) and pseudo‐cyclic electron flow (Shikanai, 2014), that were shown to be particularly important to protect PSI from over‐reduction and damage (Allahverdiyeva et al., 2015; Storti et al., 2020).

All photosynthetic organisms present multiple regulatory mechanisms, a clear indication that modulation of photosynthesis is essential. On the other hand, not all mechanisms are conserved in all organisms, and interesting differences are observed in different species, possibly in correlation with evolutionary history and colonization of specific ecological niches (Alboresi et al., 2019; Goss & Lepetit, 2015; Quaas et al., 2014). The exploration of the diversity of regulatory responses in various phylogenetic groups thus represents a valuable source of information for a better understanding of their molecular mechanisms and biological role.

In this context, microalgae are particularly interesting since they are highly diverse, with more than 200,000 estimated species colonizing a wide range of ecosystems (Bleakley & Hayes, 2017). Eukaryotic algae originated from a primary endosymbiosis event approximately 1600 million years ago (Mya), which gave origin to Rhodophyta (red algae), Chlorophyta (green algae), and Glaucophyta (Gaignard et al., 2019). Diverse clades of photosynthetic organisms, including heterokontophytes, dinoflagellates, and haptophytes, originated through secondary and tertiary endosymbioses, thus further increasing the biodiversity of these organisms. Most of these latter endosymbiosis events involved phototrophic red microalgae, which thus have a central place in the evolution of photosynthetic organisms (Keeling, 2013).

Red algae (Rhodophyta) are estimated to include more than 7000 species, consisting of both micro and macroalgae (Guiry & Guiry, 2021). According to the more recent classification, the phylum Rhodophyta is divided into two subphyla, Cyanidiophytina and Rhodophytina (Yoon et al., 2006). The former includes unicellular red microalgae living in extreme environments, such as *Cyanidioschyzon merolae* or *Galdieria sulphuraria*. Rhodophytina is, instead, divided into six classes, three of them including microalgae: Porphyridiophyceae, Rhodellophyceae, and Stylonematophyceae.

Red algae present chlorophyll *a* (Chl *a*) (Gaignard et al., 2019; Gantt et al., 2003) and β‐carotene as the major carotenoid (Schubert et al., 2006). Differently from other eukaryotes, red algae still present phycobilisomes as antenna proteins connected to PSII (Gantt et al., 2003; Marquardt & Rhiel, 1997; Wolfe et al., 1994). Like other eukaryotes, however, red algae also show transmembrane antennas LHC proteins, called LHCR, associated with Photosystem I (PSI) (Bhattacharya et al., 2013; Brawley et al., 2017; Neilson & Durnford, 2010). Even though structural biology allows obtaining high‐resolution structural models and new information on the composition of photosynthetic complexes (Antoshvili et al., 2019; Ma et al., 2020; Pi et al., 2018), the knowledge concerning the in vivo regulation of photosynthesis in red algae is still limited (Eggert et al., 2007; Kowalczyk et al., 2013; Krupnik et al., 2013; Magdaong & Blankenship, 2018).

To increase available information on the biodiversity of photosynthetic organisms and, in particular, of the red lineage, we investigated a recently identified unicellular mesophilic red alga *Dixoniella giordanoi* (Sciuto et al., 2021) belonging to the Rhodellophyceae. We assessed the response of its photosynthetic apparatus to illumination dynamics with different timescales, investigating its acclimation to different light intensities as well as the capacity of activating NPQ response, xanthophyll cycle, and effective cyclic electron transport.

## 2 MATERIALS AND METHODS

### 2.1 Culture conditions

*Dixoniella giordanoi* was isolated from Adriatic Sea samples and identified as a new species of the genus *Dixoniella* (Sciuto et al., 2021). Here it was grown in sterile‐filtered f/2 medium (Guillard & Ryther, 1962), using sea salts 32 g/L from SIGMA, 40 mM Tris HCl pH 8, SIGMA Guillard's (f/2) marine water enrichment solution ×1. Growth experiments were performed in Erlenmeyer flasks with orbital shaking, starting from a preculture at exponential phase grown at 100 μmol of photons m^−2^ s^−1^ illuminated with white LED lamps. The preculture was diluted to a final OD_750_ equal to 0.2, corresponding to approximately 0.9 × 10^6^ cells/mL in a final volume of 30 mL. OD was measured using 1 cm length cuvettes. Constant illumination of 20, 100, and 400 μmol of photons m^−2^ s^−1^ was provided with white LED lamps. The temperature was kept at 22°C ± 1°C in a growth chamber. Algal growth was maintained for 4 days and daily monitored by cell counting. Maximal growth rates (expressed as day^−1^) were calculated as the slope of the exponential phase of growth curves. For preliminary transmission electron microscope observations and Western blot analysis, algae were grown using f/2 medium enriched with nitrogen, phosphate and iron sources (0.75 g/L NaNO_3_, 0.05 g/L NaH_2_PO_4_, and 0.0063 g/L FeCl_3_ · 6H_2_O final concentrations), in 5 cm diameter Drechsel's bottles with a 250 mL working volume and bubbling of air enriched with 5% CO_2_.

### 2.2 Transmission electron microscopy (TEM)

Cells were collected by centrifugation (10 min, 17,000*g*) and fixed overnight at 4°C in 3% glutaraldehyde in 0.1 M sodium cacodylate buffer (pH 6.9) and post‐fixed for 2 h in 1% osmium tetroxide in the same buffer. The specimens were dehydrated in a graded series of ethyl alcohol and propylene oxide and embedded in Araldite. Ultrathin sections (80–100 nm) were cut with an ultramicrotome (Ultracut; Reichert‐Jung, Vienna, Austria) and stained with lead citrate and uranyl acetate; they were then analyzed under a transmission electron microscope (Tecnai G2; FEI, Hillsboro, Oregon) operating at 100 kV.

### 2.3 Pigment extraction and analysis

Chlorophyll *a* and total carotenoids were extracted from *D. giordanoi* cultures after 4 days of growth. Cells were centrifuged for 10 min at 17,000*g*. Hydrophobic pigments were extracted from centrifuged cells at 4°C using a 1:1 biomass to solvent ratio of 100% N,N′‐dimethylformamide for at least 24 h under dark conditions. Pigment concentrations were determined spectrophotometrically using extinction coefficients (Porra et al., 1989; Wellburn, 1994).

### 2.4 High‐performance liquid chromatography pigment extraction and analysis

Pigments were extracted from *D. giordanoi* cultures after 4 days of growth. Cells were harvested by 10 min centrifugation at 10,000*g* at room temperature, and the supernatant was carefully discarded. Cells were disrupted with a Mini Bead Beater (Biospec Products). Four cycles were performed: rupture at 3500 OPM for 10 s in the presence of glass beads (150–212 mm diameter) and acetone 80% followed by 30 s in ice. The extracted pigments were then centrifuged at 20,000*g* for 10 min, and the supernatant was kept for the analyses. The content of individual carotenoids was determined using an High‐performance liquid chromatography (HPLC; 1100 series, Agilent), equipped with a reversed‐phase column (5 μm particle size; 25 × 0.4 cm; 250/4 RP 18 Lichrocart) as described in Färber and Jahns (1998). The peaks of each sample were identified through the retention time and absorption spectrum (Jeffrey et al., 1997).

### 2.5 Phycobiliprotein extraction and analysis

Phycobiliproteins were extracted from *D. giordanoi* cultures after 4 days of growth. Cells were harvested by 10 min centrifugation at 10,000*g* at room temperature and washed twice in phosphate buffer (0.01 M Na_2_HPO_4_ and 0.15 M NaCl). Cell rupture was performed by three freeze–thaw cycles in liquid nitrogen in dark conditions to avoid phycobiliprotein photodegradation. Disrupted cells were resuspended in phosphate buffer (1:1), and phycobiliprotein concentrations were determined spectrophotometrically as described in Bennett and Bogobad (1973).

### 2.6 SDS‐PAGE electrophoresis and Western blotting

Samples were collected from cultures in the late exponential phase. Cells were disrupted with a Mini Bead Beater (Biospec Products, Oklahoma) at 3500 rpm for 20 s in the presence of glass beads (150–212 mm diameter), B1 buffer (400 mM NaCl, 2 mM MgCl_2_, and 20 mM Tricine–KOH, pH 7.8), 0.5% milk powder, 1 mM PMSF, 1 mM DNP‐e‐amino‐n‐caproic acid, and 1 mM benzamidine. The ruptured cells were then solubilized in a buffer (×3) contained 30% glycerol, 125 mM Tris, pH 6.8, 0.1 M dithiothreitol, and 9% SDS at RT for 20 min. SDS‐PAGE analysis was performed with a Tris‐glycine buffer system as previously described (Laemmli, 1970) with acrylamide at a final concentration of 12%. Western blot analysis was performed by transferring the proteins to nitrocellulose (Bio Trace, Pall Corporation, Auckland, New Zealand) and detecting them with alkaline phosphatase‐conjugated antibodies. The antibodies recognized the PSI subunits PsaA and PsaD (Agrisera), D2, LHCX1, VCP, and LHCII proteins (antibodies produced by immunizing New Zealand rabbits with purified spinach protein [D2, LHCII] or recombinant *N. gaditana* proteins [VCP and LHCX1]; Meneghesso et al., 2016).

### 2.7 In vivo monitoring of photosynthetic parameters

Chlorophyll fluorescence was determined in vivo using Dual PAM 100 from Waltz. The parameters Fv/Fm, NPQ, and Y(II) were estimated using a light curve protocol, where the cells were stepwise exposed to increasing light intensity every 1 min, from 0 to 2006 μmol of photons m^−2^ s^−1^, after 20 min of dark adaptation. Fv/Fm, NPQ, and Y(II) were calculated as (Fm − F_0_)/F_0_, (Fm − Fm′)/Fm′, and (Fm′ − F)/Fm′, respectively (Walz, 2006). Far‐red light was switched on during the measurements unless otherwise stated. When actinic light was set off, NPQ was calculated only by a series of saturating pulses (6000 μmol of photons m^−2^ s^−1^, 600 ms) every 1 min. NPQ was also estimated by incubating cells with nigericin (10 μM) for 10 min before the measure. The PSII antenna size was measured according to the fluorescence induction kinetics using a JTS‐10 spectrophotometer in the fluorescence mode. Two milliliters of samples with a final concentration of 5 × 10^6^ cell/mL were incubated with DCMU (3,4‐dichlorophenyl‐1,1‐dimethylurea, 10 μM) for 10 min after 20 min of dark adaptation. The induction kinetics was measured upon excitation with 80 or 150 μmol of photons m^−2^ s^−1^ of actinic light at 630 nm. In the presence of this inhibitor, an average of 1 photon per PSII center is absorbed at time *t*, corresponding to 2/3 of the fluorescence increase. This parameter was estimated to evaluate the number of absorbed photons by photosystems II, i.e. the antenna size. The electron flows were estimated, measuring P_700_ absorption at 705 nm in intact cells. The analysis was carried out exposing cells (at ~5 × 10^6^ cells/mL) to saturating actinic light (2050 μmol of photons m^−2^ s^−1^, 630 nm) for 15 s to maximize P_700_ oxidation and reach a steady state. P_700_^+^ re‐reduction in the dark kinetics were then monitored after the light was switched off. The total electron flow (TEF) was estimated measuring the P_700_^+^ re‐reduction rates in untreated cells (Meneghesso et al., 2016; Simionato et al., 2013). The same procedure was repeated in samples treated with DCMU (10–80 μM) to evaluate the contribution of cyclic electron flow (CEF) and with DCMU in combination with DBMIB (dibromothymoquinone, 100–300 μM). In all cases, re‐reduction kinetics of P_700_^+^ were quantified as the rate constant 1/*τ* after fitting with a single exponential. By multiplying these rate constants the fraction of P_700_ oxidized (which was obtained by comparison with DCMU and DBMIB‐poisoned cells), the number of electrons transferred per unit of time was evaluated.

### 2.8 Oxygen evolution

The evolution of oxygen through increasing light intensities was measured using the O2k FluoroRespirometer (Oroboros Instruments; Doerrier et al., 2018). The measuring chambers were magnetically stirred at 750 rpm, and the oxygen concentration of the chambers was measured with a frequency of 2 s. The light source was a blue LED (emitting wavelength range 439–457 nm with the peak at 451 nm) attached to the chamber of the instrument (provided by Oroboros Instruments, manufactured by Osram Oslon). The 2 mL chambers were filled with a growth medium containing 5 mM NaHCO_3_ and let equilibrate to experimental temperature (22°C). Then, a small fraction of the medium was replaced with an aliquot of the cell suspension to reach a final concentration in the chamber of 0.5 × 10^6^ cells/mL. The chambers were then closed, and the oxygen consumption rate at dark was calculated. The light was turned on at 25 μmol of photons m^−2^ s^−1^ until stabilization of the oxygen flow (5–10 min). This was done recursively for the following light intensities: 25, 50, 100, 150, 200, 250, 300, 400, 500, 600, 700, 800, 900, and 1000 μmol of photons m^−2^ s^−1^. The reported values of oxygen evolution rates at each intensity correspond to the median of 40–50 points in the stable region of oxygen flow minus the oxygen flow at dark (respiration).

## 3 RESULTS

### 3.1 Photosynthetic apparatus composition in *Dixoniella giordanoi*

**FIGURE 1:**
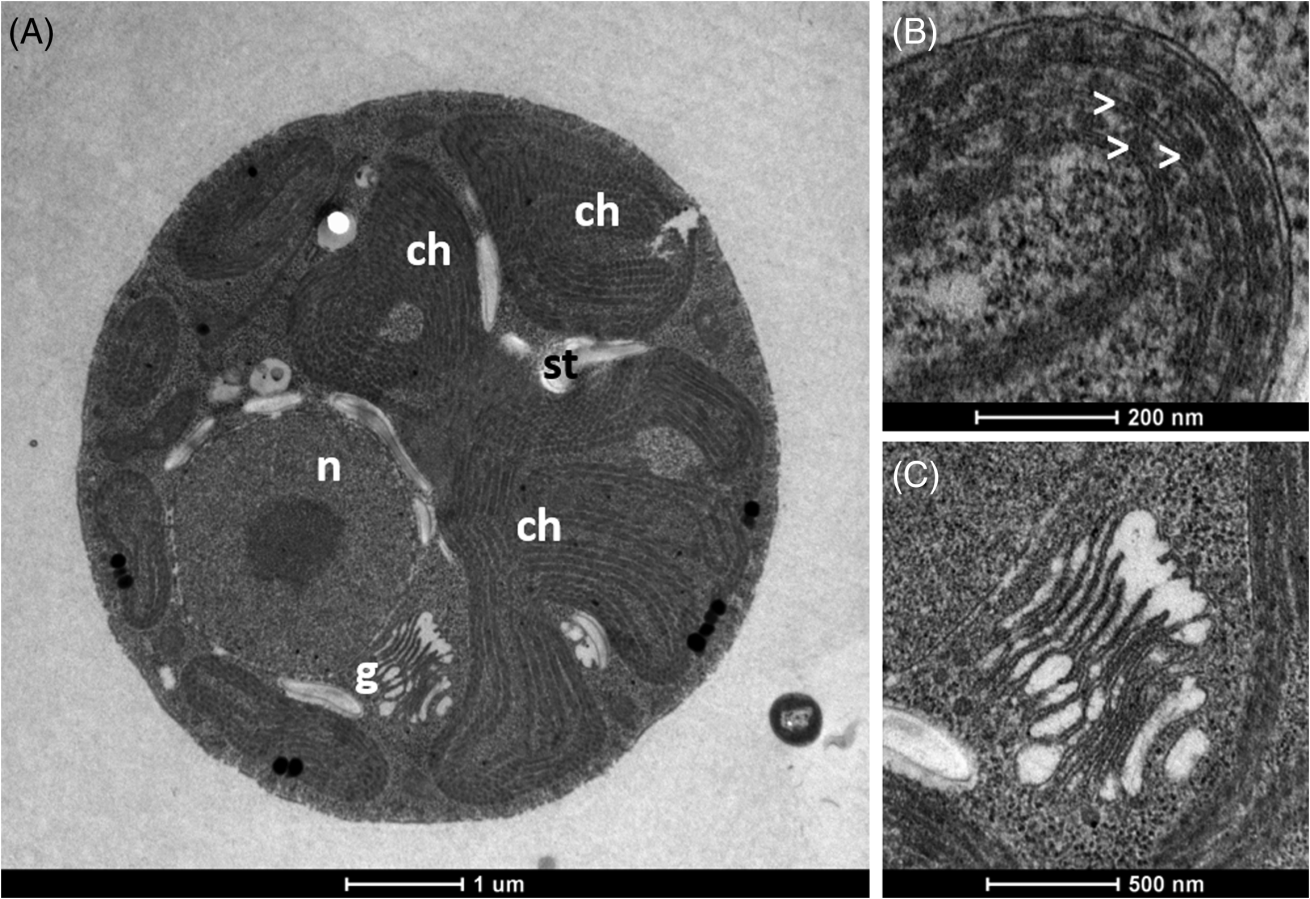
Transmission electron microscopy of *D. giordanoi* cells. (A) Complete view of a cell. Specific features and organelles are marked as n, nucleus; g, Golgi apparatus; ch, chloroplast; st, starch. (B,C) show details of thylakoids and dictyosomes of the Golgi apparatus, respectively. The white arrows indicate phycobilisomes associated with the thylakoids

*Dixoniella giordanoi* cells highlighted the presence of a single chloroplast characterized by a multilobate shape, occupying the largest part of cell volume, as typically found in red algae (Figure 1A). Within the chloroplasts, the thylakoids are unstacked for the presence of disk‐shaped multiple phycobilisomes, present with an alternated arrangement on the thylakoid membranes (Figure 1B). Starch granules were visible outside of the chloroplast. Indeed, differently from green algae and plants, red algae carbohydrate reserves, called floridean starch, are found in the cytoplasm and not in the organelles (Viola et al., 2001). An eccentric nucleus with associated Golgi dictyosomes was also visible (Figure 1C). The peculiar position of Golgi bodies near the outer membrane of the nuclear envelope is indeed a characteristic feature of only a few red algae genera, like *Dixoniella*, *Glaucosphaera*, and *Neorhodella* (Scott et al., 2011).

**FIGURE 2:**
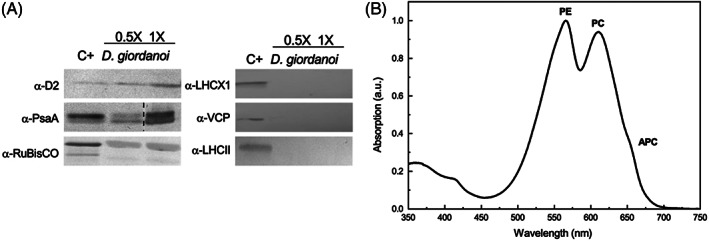
Composition of *D. giordanoi* photosynthetic apparatus. (A) Western blotting targeting different components of the photosynthetic apparatus from PSII (D2), PSI (PsaA), RuBisCO, and antenna complexes (LHCX1, VCP, and LHCII). Different dilutions of total cell extracts were loaded. The ×1 corresponds to 1 μg of Chl for each *D. giordanoi* sample, except for the case of PsaA, where 2 μg were loaded. As positive control (C+) total proteins extracted from the moss *Physcomitrella patens* were loaded for the targeting of D2, PsaA, RuBisCO, and LHCII. Extracts from *Nannochloropsis gaditana* were used as a positive control for LHCX1 and VCP. The dashed line indicates the removal of a lane not essential for the picture. (B) Absorption spectrum of isolated PBS fraction. Three absorption peaks are identified as phycoerythrin (PE), phycocyanin (PC), and allophycocyanin (APC) from their absorption maximum

Western blot analysis performed to evaluate the composition of the photosynthetic apparatus allowed the detection of PsaA and D2 proteins, core subunits of, respectively, PSI and PSII even using antibodies raised against green algae and plants proteins, respectively, confirming the conservation of the sequences of these multiprotein complexes in photosynthetic organisms (Figure 2A). The presence of RuBisCO was also detected, corroborating the high conservation of the protein sequence. The presence of transmembrane antennas LHC protein was also tested using antibodies raised against proteins from highly divergent proteins, namely LHCX1 and VCP antenna from the heterokont *Nannochloropsis gaditana* and LHCII from plants. In neither case, there was any recognition in *D. giordanoi* samples confirming that, unlike the core complex of photosystems, antenna complexes show larger variability among the different groups of photosynthetic organisms.

Different protocols available in the literature were tested and optimized to isolate a phycobilisome (PBS) enriched soluble fraction from hydrophobic thylakoids. Even though the PBSs were not completely purified, absorption analysis of the soluble protein fraction revealed the presence of three distinct absorption peaks identifiable as typical of phycoerythrin (PE, peak at 562 nm), phycocyanin (PC, 615 nm), and allophycocyanin (APC, 652 nm), respectively (Figure 2B).

### 3.2 Response of *Dixoniella giordanoi* to growth under different light conditions

**FIGURE 3:**
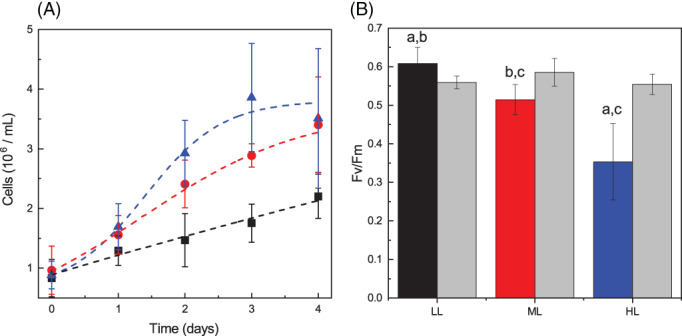
*D. giordanoi* growth under different light regimes. (A) Growth curve for *D. giordanoi* cells exposed for 4 days to LL, ML, and HL (20, 100, and 400 μmol of photons m^−2^ s^−1^), shown as black squares, red circles, and blue triangles, respectively. The fitting of experimental data is shown as dashed lines. Average ± sd are reported (n > 4). (B) Photosystem II quantum yield quantified from Fv/Fm, of *D. giordanoi* after 4 days of growth in LL, ML, and HL (black, red, and blue, respectively). a, b, and c indicate differences statistically significant from ML, HL, and LL, respectively (one‐way ANOVA, *p* < 0.05, n > 4, ±sd). Fv/Fm values after 24 h of dark recovery are represented with grey bars (n = 3, ±sd)

In nature, the light intensity is highly dynamic, with changes that can occur with different kinetics spanning from seconds to weeks. To cope with light variability, photosynthetic organisms evolved multiple regulatory mechanisms with different activation timescales, enabling responses to short‐term and long‐term variations. Here *D. giordanoi* ability to respond to different illumination was assessed by exposing batch cultures to three light intensities of 20, 100, and 400 μmol of photons m^−2^ s^−1^, hereafter called LL, ML, and HL (Low, Medium, and High Light), respectively. Cultures exposed to LL showed the lowest growth rate, equal to 0.22 ± 0.03 day^−1^, suggesting that this light intensity was limiting (Figure 3A). Indeed, cells exposed to ML showed faster growth (0.46 ± 0.07 day^−1^). A further four times increase in light intensity yielded a further small increase in growth rate (0.60 ± 0.05 day^−1^), suggesting that light saturation was reached.

To assess how the photosynthetic apparatus responded to different light intensities, the maximum PSII quantum yield was estimated by Fv/Fm. (Figure 3(B); Murata et al., 2007). PSII quantum yield was 17% and 38% lower in ML and HL compared with LL. If cells were left in the dark for 24 h, the Fv/Fm completely recovered in all cases, suggesting that the lower values were attributable to the photoinhibition of PSII experienced at ML and especially HL that is recovered, allowing enough time for damage repair.

### 3.3 Acclimation of *Dixoniella giordanoi* to different illumination

Photosynthetic organisms exposed to different light regimes often respond by modulating the photosynthetic apparatus composition and functionality. Indeed, chlorophyll (Chl) content in *D. giordanoi* was strongly altered by growth under different illumination regimes. Chl content was higher in LL cells with approximately 40% and 70% lower content in ML and HL, respectively (Table 1). The relative content of carotenoids, on the contrary, increased with the light intensity, as shown by the increase of Carotenoid/Chlorophyll ratio (Car/Chl), suggesting the relatively increased accumulation of these pigments with antioxidant activity. HPLC analysis showed that zeaxanthin and β‐carotene are the main carotenoids in *D. giordanoi* in all light conditions. The relative content of zeaxanthin was progressively higher in cultures exposed to higher light intensity, while β‐carotene remained stable.

**TABLE 1:** Pigment composition of *D. giordanoi* cells grown under different light regimes

	LL	ML	HL
Chl *a* content (pg/cell)	1.01 ± 0.17^a^	0.57 ± 0.13^b^	0.32 ± 0.13^b^
Car/Chl ratio (pg/pg)	0.41 ± 0.03^a^	0.61 ± 0.09^b^	0.78 ± 0.08^b^
Zea/Chl *a* (mol)	0.28 ± 0.01^a^	0.43 ± 0.09^b^	0.67 ± 0.14^b^
Beta/Chl *a* (mol)	0.21 ± 0.01	0.19 ± 0.10	0.29 ± 0.06
Phycocyanin (pg/cell)	3.11 ± 0.90^a^	0.74 ± 0.25^b^	0.19 ± 0.16^a^ ^,^ ^b^
Allophycocyanin (pg/cell)	0.64 ± 0.35	0.14 ± 0.04	n.d.
Phycoerythrin (pg/cell)	1.37 ± 0.46^a^	0.28 ± 0.11^b^	n.d.

*Note:* Pigment composition of *D. giordanoi* cells assessed after 4 days of growth at LL, ML, and HL (20, 100, and 400 μmol of photons m^−2^ s^−1^). Chl content per cell, Car/Chl ratio, and phycobiliproteins (phycocyanin, allophycocyanin, and phycoerythrin) content per cell are reported (n = 3). n.d., not detectable for values smaller than 0.05 pg/cell.

a and b indicate statistically differences from ML and LL samples, respectively (one‐way ANOVA, *p* < 0.05, n = 3, ±sd).

Phycobilisomes (PBS) content was also strongly affected by light intensity, as evidenced by the decrease by 75%–80% in ML cells compared with LL for all three pigments classes: phycocyanin, allophycocyanin, and phycoerythrin. The decrease was even larger in HL cells, which showed a 95% lower content of PBS than LL acclimated cells (Table 1). Taken together, these data indicate that *D. giordanoi* showed a strong acclimation response with modulation of Chl, carotenoids, and PBS content.

**FIGURE 4:**
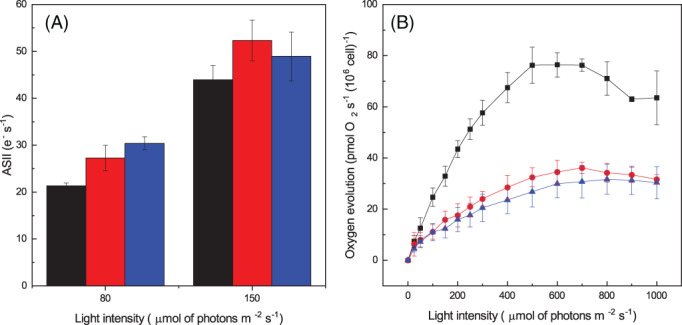
Acclimation of *D. giordanoi* cells to different light illumination. (A) Average PSII antenna size for all samples. Fluorescence measurements were taken with 10 million DCMU‐treated cells in the presence of 80 or 150 μmol of photons m^−2^ s^−1^ of actinic light at 630 nm. (B) Oxygen evolution activity of acclimated cells exposed to increasing light intensity. Measurements were taken with 1 million cells. For both the pictures, LL, ML, and HL cells are represented in black, red, and blue, respectively. Data are reported as the average of three biological replica ±sd

The assessment of functional PSII antenna size using red light at 630 nm, thus poorly absorbed by PBS, showed no major differences between LL, ML, and HL cells, suggesting that LHC content was not significantly regulated (Figure S1; Figure 4A).

Acclimation of photosynthetic activity was further assessed by measuring the oxygen evolution activity in cells exposed to increasing light intensities (Figure 4B). Comparing data measured with an equal cell concentration, with the lowest light intensities, LL cells showed a higher oxygen evolution activity compared with ML and HL, suggesting a higher light harvesting efficiency of these cells. Since blue excitation light is not well absorbed by PBS, the difference is likely underestimated. Oxygen evolution activity increased until reaching saturation at 500 μmol of photons m^−2^ s^−1^ and showed a decrease, likely because of photoinhibition, at higher light intensities. ML and HL cells instead reached saturation at higher illumination (7–800 μmol of photons m^−2^ s^−1^), and photosynthetic activity did not decrease if the light was further increased.

All cells showed a similar oxygen evolution capacity per Chl amount (Figure S2), suggesting all cells, including HL cells have similar photosynthetic activity. This observation also suggests a similar light harvesting efficiency of Chl binding pigments, consistent with antenna size estimations (Figure S2).

**FIGURE 5:**
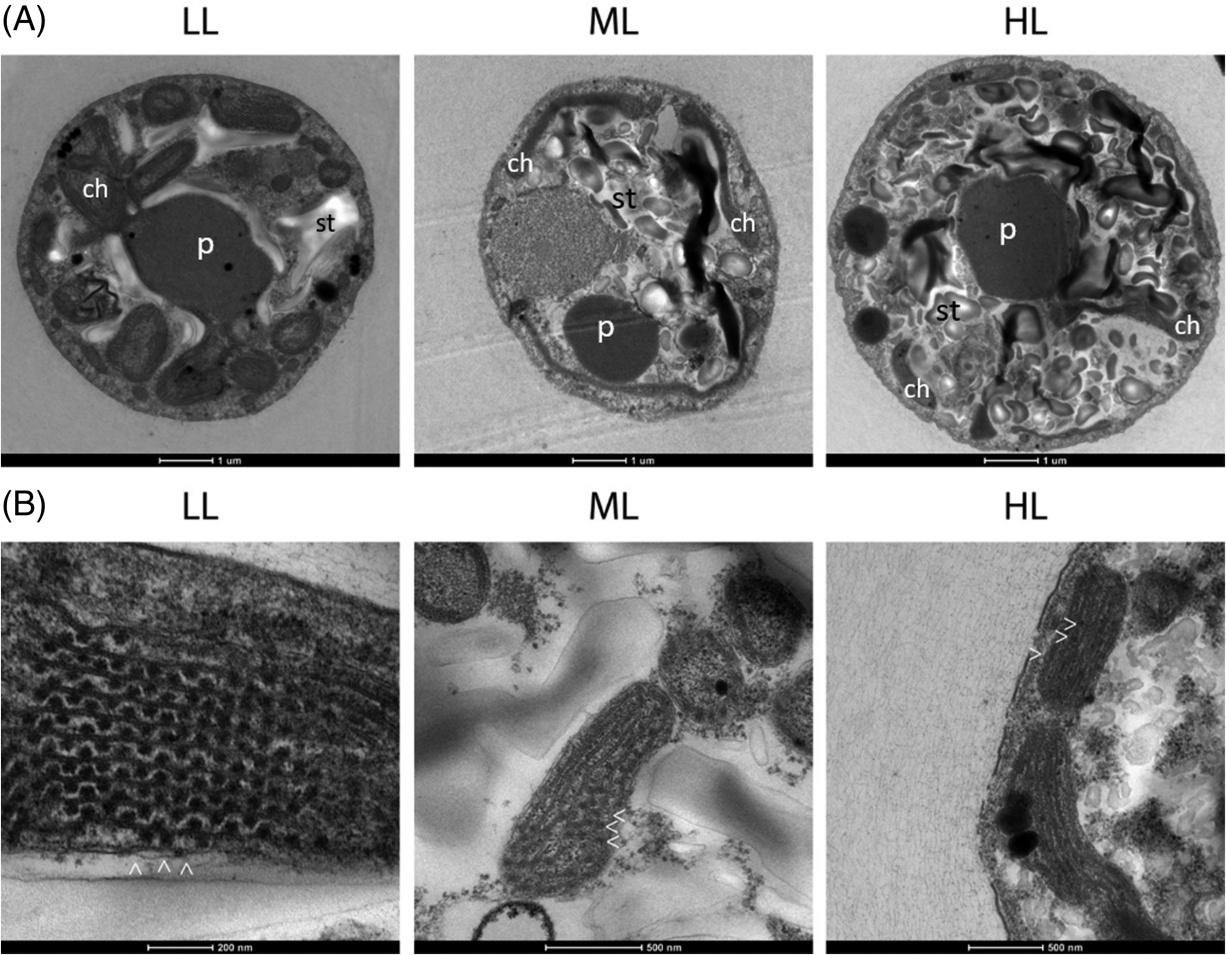
Ultrastructure of *D. giordanoi* cells acclimated to LL, ML, and HL. Panel (A) shows images of the whole cells. Specific features and organelles are marked as p, pyrenoid; ch, chloroplast lobes; st, starch. In (B) details of thylakoid membranes are shown. The white arrows indicate phycobilisomes associated with the thylakoids

The effect of light acclimation on *D. giordanoi* cell ultrastructure was assessed using transmission electron microscopy (Figure 5). Thylakoid membrane content and their organization were strongly affected by light intensity. In LL cells, thylakoid membranes were arranged in parallel arrays that included regularly spaced phycobilisomes. In ML cells and, to an even larger extent, in HL cells, thylakoids showed drastically reduced size, occupying a smaller part of cell volume, and were less organized. The number of phycobilisomes was also clearly lower in ML than in LL, and only a few were visible in HL cells. ML and especially HL cells also showed a large part of cell volume occupied by starch reserves. This indicates that an increasing fraction of carbon fixed was converted into reserve carbohydrates suggesting that photosynthetic activity was in excess in comparison to cells' ability to use light energy for growth, with other factors becoming limiting. The comparison of LL cells with ML and HL also showed a progressive reduction of chloroplast lobes volume, while the pyrenoid, containing Rubisco, remained well visible, consistent with a decrease in light harvesting efficiency while maintaining carbon fixation capacity.

### 3.4 Photosynthesis regulatory mechanisms in *Dixoniella giordanoi*

**FIGURE 6:**
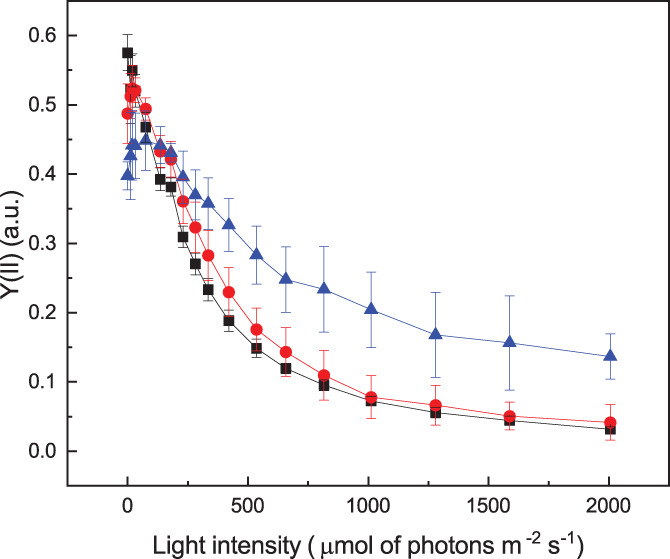
Photosynthetic regulation of *D. giordanoi* to different light intensities. Chl fluorescent kinetics were used to calculate PSII quantum yield expressed as Y(II) of acclimated cells and illuminated with a progressively stronger light. LL, ML, and HL cells are represented in black, red, and blue, respectively. During the measurements, the far‐red light was switched on. Data are expressed as the average of three biological replica ±sd

Photosynthetic organisms modulate not only the composition but also the activity of the photosynthetic apparatus in response to the illumination regimes. Photochemical capability can be deducted by measuring the effective quantum yield of PSII, Y(II), in cells illuminated with an actinic light intensity of different intensity. As shown in Figure 6, Y(II) progressively decreased with the increase of illumination because of the increased saturation of photochemical capacity. While Y(II) dropped rapidly in cells grown in LL and ML, HL cells maintained photochemical capability under strong illumination and did not show complete saturation even with the strongest illumination tested, showing that *D. giordanoi* cultures acclimated to high light can exploit a large fraction of absorbed light for photochemistry even under strong illumination.

**FIGURE 7:**
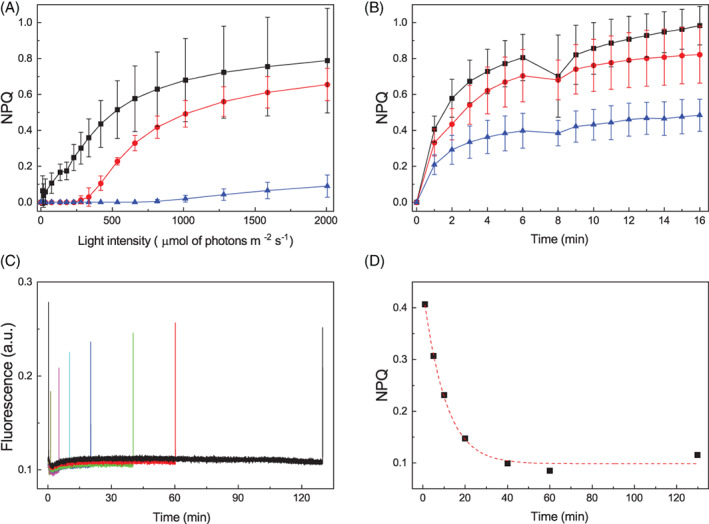
Non photochemical quenching in *D. giordanoi*. (A) NPQ was measured exposing acclimated cells to increasing light intensity. During the measurements, the far‐red light was switched on. (B) NPQ of acclimated cells exposed to saturating pulses every minute with actinic light off (far‐red off). For both figures, LL, ML, and HL cells are represented in black, red, and blue, respectively. Data are shown as the average of three biological replica ±sd. (C) Fluorescence traces of LL cells illuminated by a first saturating pulse followed by a second one after 1, 5 10, 20, 40, 60, or 130 min (in dark yellow, magenta, cyan, blue, green, red, and black, respectively). (D) The graph reports the kinetics of NPQ relaxation after one saturating pulse. The fitting of experimental data is shown as a dotted line

Fluorescence measurements can also be exploited to assess regulatory mechanisms of photosynthesis and, for instance cell ability to activate thermal dissipation of excess energy quantifiable as NPQ. Figure 7(A) shows that *D. giordanoi* LL cells can activate a measurable NPQ when exposed to illumination of different intensities, but this capacity is lower in ML cells and even further decreased in HL cells. The addition of nigericin, a molecule dissipating membrane ΔpH, does not inhibit NPQ in *Dixoniella*, suggesting the response is not pH‐dependent (Figure S3).

Earlier reports showed in other red microalgae that saturating pulses alone could induce a detectable NPQ (Delphin et al., 1998). Indeed, *Dixoniella* cells simply exposed to a series of saturating pulses with the actinic light off showed the progressive decrease of fluorescence maxima and thus an apparent NPQ, that is not significantly different from the response with the light on (Figure 7(B); Figure S4). A single saturating pulse is indeed sufficient to induce an NPQ value equal to 0.4 (Figure 7B), which takes a significant over 40 min, to be relaxed (Figure 7(C,D)). Overall, the results show that an apparent NPQ is activated in *D. giordanoi*, but it has some peculiar characteristics different from those normally observed in other algae and plants.

### 3.5 Regulation of photosynthetic electron transport

**FIGURE 8:**
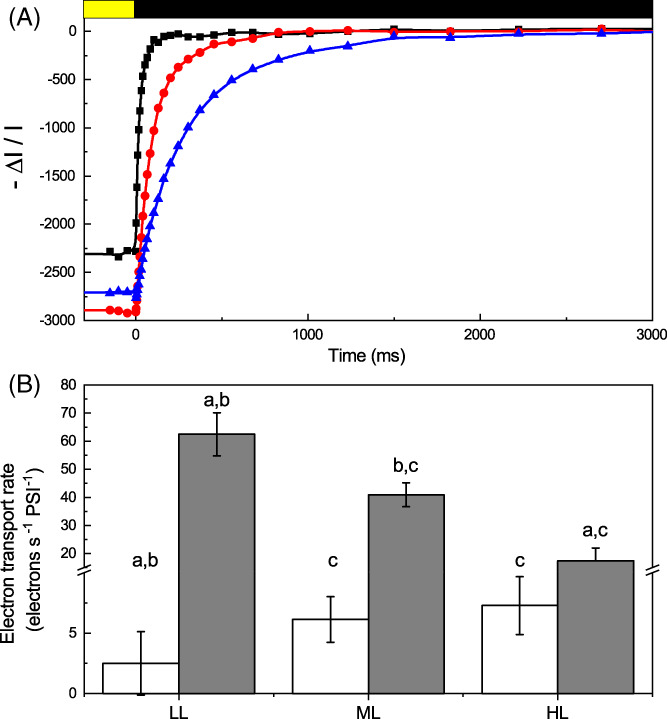
Regulation of photosynthetic electron transport in *D. giordanoi*. (A) Representative P_700_ redox kinetics from cells grown in ML. Cells acclimated to 100 μmol of photons m^−2^ s^−1^ (ML) were exposed to saturating light intensity (2050 μmol of photons m^−2^ s^−1^) for 15 s before the dark recovery kinetics. Reduction of P_700_^+^ after switching light off is followed by monitoring differential absorption at 705 nm. Untreated cells, cells treated with DCMU, and cells treated with DCMU+DBMIB are shown as black squares, red circles, and blue triangles, respectively. (B) Rates of cyclic electron flow (CEF, white) and total electron flow (TEF, gray) were evaluated from P_700_^+^ reduction kinetics after illumination in LL, ML, and HL acclimated cells. a, b, and c indicate differences statistically significant from ML, HL, and LL, respectively (one‐way ANOVA, *p* < 0.05, n = 3, ±sd)

Chlorophyll fluorescence signal in intact cells primarily originates from PSII, and thus, the previous analyses are mainly indicative of its activity and regulation. PSI activity can instead be assessed by monitoring the P_700_ redox state in vivo. To this aim, cells were first exposed to actinic light inducing oxidation of PSI reaction center, generation of P_700_^+^ that can be monitored from a differential absorption signal at 705 nm (Simionato et al., 2013). After cells were exposed to illumination for 5 min and reached steady‐state photosynthesis, the light was switched off, allowing for the reduction of P_700_^+^. The kinetics of P_700_ reduction depends on the rate of electron transports from PSII and cytochrome b_6_f to PSI and thus allows estimating the total electron flow (TEF), i.e. the sum of all the electron transport processes through PSI. The same measurements were repeated in the presence of inhibitors of PSII and cytochrome b_6_f (DCMU and DBMIB, respectively) to assess the relative contribution of the linear (LEF) and cyclic (CEF) electron pathways to this total electron transport. As shown in Figure 8(A), PSII inhibition through DCMU strongly reduced electron transport, suggesting that linear electron transport through PSII and PSI is the main pathway in *D. giordanoi*, as commonly observed in various photosynthetic organisms. Treatment with both DCMU and DBMIB, thus inhibiting both PSII and Cyt b_6_f, showed a further decrease in P_700_ reduction kinetics, suggesting that there is a significant contribution of cyclic electron flow around PSI to electron transport (Figure 8A). Similar experiments were repeated for cells acclimated to different conditions to assess the contribution of linear and cyclic electron transport to photosynthesis. Cells exposed to higher light intensities showed a small decrease in linear electron transport capability, but this was at least partially compensated by a relative increase of cyclic electron transport, whose activity was stronger in high light acclimated cells (Figure 8B).

## 4 DISCUSSION

### 4.1 Acclimation to different illumination by modulation of Chl and phycobilisomes content

Photosynthesis involves a complex set of redox reactions occurring in an oxygen‐rich environment that are prone to the generation of reactive species. Many environmental parameters like temperature, irradiance, CO_2_, and nutrients availability strongly influence photosynthesis requiring a continuous modulation. To thrive in such a dynamic and complex environment, photosynthetic organisms evolved multiple regulatory mechanisms to maintain photosynthetic efficiency while also protecting from the danger of over‐excitation and over‐reduction. Functional genomic studies with model organisms, *Arabidopsis thaliana* and *Chlamydomonas reinhardtii* in particular, allowed isolation and characterization of specific mutants resulting in the identification of several genes involved in the regulation of photosynthesis (Eberhard et al., 2008; Li et al., 2009). Mutant generation can, however, also drive to pleiotropic or compensatory effects that may impair a full understanding of biological function. A clear example can be found in the case of cyclic electron transport that depends on multiple mechanisms with significant functional overlap and where single mutants generally have mild phenotypes, leading to an underestimation of their biological relevance. On the contrary, when multiple mutations are combined, phenotypes are much more severe, showing how regulation of electron transport is indeed essential for photosynthesis (Munekage et al., 2004; Storti et al., 2020).

While all photosynthetic organisms present several regulatory mechanisms modulating activity, they are not all conserved in all species. Exploring biological diversity is thus a powerful approach to identify organisms showing specific combinations of regulatory mechanisms or unexpected features (Berne et al., 2018), complementing functional genetic studies. Algae with their large biodiversity are particularly interesting in this context since they are a source of variability that can be exploited for the understanding of the distribution of regulatory mechanisms (Shimakawa et al., 2018), motivating the analysis of under‐investigated groups like the mesophilic red algae studied here.

*D. giordanoi* cultures under different light intensities respond to environmental conditions through the reorganization of the photosynthetic apparatus and the modulation of its photosynthetic efficiency (Figure 3B). *D. giordanoi* cells cultivated under high light intensity show a decrease in the cell Chl content and a reduction of the number of thylakoid membranes (Table 1; Figure 4), a commonly observed response (Falkowski & LaRoche, 1991; Walters, 2005). *D. giordanoi* also shows a strong modulation of the content of PBS that in HL acclimated cells decrease by 95% and become barely detectable (Table 1; Figure 5). Such modulation is much more extreme of what was observed for LHC antennas both in plants and algae that even upon exposure to extreme illumination retain a relatively large antenna system (>50%; Ballottari et al., 2007; Bonente et al., 2012; Meneghesso et al., 2016). The light‐harvesting phycobilisomes are important to increase light absorption under low light intensities and, because of their presence, red algae can efficiently harvest radiation between 490 and 650 nm. This ability represents a significant competitive advantage in environments characterized by low sunlight availability, such as deep waters (Larkum, 2016). On the other hand, in case of exposition to strong illumination, the presence of PBS becomes detrimental, pushing *D. giordanoi* cells to almost remove the PBS antenna system completely.

Red algae antenna system also includes transmembrane LHC, as in other eukaryotes. The functional analysis of the antenna size (Figure 4A) and the oxygen evolution activity normalized per Chl content (Figure S2) using wavelengths poorly absorbed by PBS suggest that the LHC content is instead not significantly modulated between LL, ML, and HL cells. This suggests that acclimation response is dominated by two main responses: the modulation of the overall number of photosynthetic complexes and thus the Chl content per cell the regulation of PBS accumulation, while the LHC/reaction center ratio remains rather constant.

### 4.2 *Dixoniella giordanoi* activates a peculiar fluorescence quenching as other unicellular red algae

NPQ consists of the thermal dissipation of excess energy and it is a regulatory mechanism widespread in photosynthetic organisms. Plants and many species of algae can activate NPQ thanks to specific proteins, PSBS or LHCX (Li et al., 2000; Peers et al., 2009). An NPQ mechanism with different properties is present in cyanobacteria as well, although it depends on the presence of another protein, OCP (Kirilovsky & Kerfeld, 2016). No gene encoding for these proteins has been found in red microalgae genomes sequenced so far, thus questioning the presence of an NPQ response in these organisms.

Here we show that it is possible to observe a fluorescence quenching in *D. giordanoi* (Figure 7A), but with peculiar properties compared with what is observed in plants, other eukaryotic algae, or even cyanobacteria. This response is detectable in *D. giordanoi* independently from the presence of actinic light and it is induced by the simple application of saturating flashes during the measurement (Figure 7B). A similar phenomenon was previously observed in other red algal species (Delphin et al., 1998), suggesting this is a specific feature of at least multiple species of this phylogenetic group.

The effect of the inhibitor nigericin suggests this NPQ is not dependent on the generation of a ΔpH (Figure S3). While inhibitors' activity with unknown species must be taken with caution, this hypothesis is fully consistent with the observation that quenching induced by a single saturation pulse takes at least 40 min to relax (Figure 7D). The metabolic effect of a single saturation pulse (6000 μmol of photons m^−2^ s^−1^ for 600 ms) is expected to be negligible but, even if this is not the case, any eventual ΔpH generated should be fast consumed and cannot last for several minutes. On the contrary, the long resilience of this effect must be attributed to some signaling and regulatory phenomena activated by light but independent from the metabolic effect.

Mechanistically, one possible explanation for this quenching would be the functional disconnection of some PBS from the PSII, which would cause a reduction in absorption and thus a potential decrease in fluorescence emission signal. A light‐induced dissociation of PBS from PSII has been observed in the red alga *Porphyridium cruentum* by single‐molecule spectroscopy (Liu et al., 2008) and, indeed, PBS mobility has been suggested to be a typical feature of mesophilic red algae, differently from extremophiles (Krupnik et al., 2013). This hypothesis would be consistent with the observation that the quenching response is smaller in HL cells, where PBS content is also reduced.

Alternative hypotheses such as an NPQ activated in the reaction center, as suggested for the extremophilic red alga *Cyanidioschyzon merolae* (Krupnik et al., 2013), or the modulation of excitation energy spillover between photosystems (Kowalczyk et al., 2013) are, however, possible.

### 4.3 *Dixoniella giordanoi* accumulates zeaxanthin under strong illumination without a xanthophyll cycle

*D. giordanoi*, like other red algae, accumulates zeaxanthin together with β‐carotene (Table 1—Schubert et al., 2006; Serive et al., 2017). However, there are no other detectable xanthophylls, and violaxanthin and antheraxanthin are missing, suggesting the absence of a xanthophyll cycle. This is consistent with findings from other red algae (Marquardt, 1998; Schubert et al., 2006; Serive et al., 2017) and with the available knowledge on red algae genomes that do not contain the genes coding for the enzymes involved in the xanthophyll cycle, violaxanthin de‐epoxidase (VDE) and zeaxanthin epoxidase (ZE) (Bhattacharya et al., 2013; Coesel et al., 2008; Nozaki et al., 2007; Weber et al., 2004). Functional ZE genes have been identified in *Compsopogon coeruleus*, *Adagascaria erythrocladioides*, and *Calliarthron tuberculosum*, but they are all red seaweeds and the corresponding gene was never identified in unicellular organisms. Furthermore, these red algal ZE introduces only a single epoxy group into zeaxanthin, yielding antheraxanthin instead of violaxanthin (Dautermann & Lohr, 2017).

While the xanthophyll cycle is absent in *D. giordanoi*, data from pigment composition of cells grown under different illumination show that zeaxanthin is specifically over‐accumulated in response to strong illumination, while β‐carotene content remained stable (Table 1). Since the proteins potentially binding these carotenoids, LHC, are not increased in HL cells, these additional zeaxanthin molecules are likely found free in the thylakoid membranes. The accumulated zeaxanthin likely contributes to protection from an excess illumination in *D. giordanoi* cells acclimated to HL, similarly to what was observed in plants where zeaxanthin free in the thylakoid membranes is acting as an antioxidant, scavenging ROS (Havaux et al., 2007).

The xanthophyll cycle enables the modulation of zeaxanthin accumulation within minutes depending on illumination conditions. While *D. giordanoi* can still modulate the accumulation of zeaxanthin, the kinetics of synthesis/degradation is much slower. If cells acclimated to HL are exposed back to lower illumination it can be expected that they will retain the zeaxanthin, negatively impacting the light use efficiency. While the accumulation of zeaxanthin likely contributes to the protection from an excess of illumination in *D. giordanoi*, the absence of a xanthophyll cycle can represent a disadvantage when growing conditions are dynamic, impairing its fast synthesis and degradation when light intensity changes. At least in plants, it has been shown that this capacity for fast modulation of xanthophyll composition has a strong impact on photosynthetic productivity in a variable environment (Kromdijk et al., 2016).

### 4.4 Cyclic electron flow in Dixoniella giordanoi is increased under strong illumination

*D. giordanoi* cells show a significant capability for cyclic electron flow, which is also increased in cells acclimated to HL (Figure 8). This suggests that modulation of photosynthetic electron transport is playing a role in the *D. giordanoi* response to strong illumination. In plants, cyclic electron flow is particularly important to protect PSI from over‐reduction (Tiwari et al., 2016), while NPQ and xanthophyll cycle are more important for limiting damage from over‐excitation to PSII (Peers et al., 2009; Tian et al., 2019). *D. giordanoi* cells show signs of PSII photoinhibition, as evidenced by the lower Fv/Fm (Figure 3B) if exposed to high or even intermediate light intensity (HL/ML). Despite this damage, however, ML and HL cells are still growing much faster than LL cells that show higher PSII activity. This suggests that PSI in *D. giordanoi* is well protected, likely with the contribution of cyclic electron transport, and capable of maintaining its activity. The loss of PSII activity is thus at least partially compensated by cyclic around PSI and this is sufficient to support a fast growth (Larosa et al., 2018).

## AUTHOR CONTRIBUTION

**Nicolò Fattore:** Performed most experiments, analyzed the data, and wrote the article. **Simone Savio:** Performed most experiments, analyzed the data. **Antoni M. Vera‐Vives:** Performed oxygen evolution measurements. **Mariano Battistuzzi:** Performed HPLC analyses. **Isabella Moro:** Performed TEM analyses. **Nicoletta La Rocca:** Analyzed the data. **Tomas Morosinotto:** Designed the research, analyzed the data, wrote the article. All authors reviewed the manuscript.

## Supporting information


**Figure S1** Modulation of the functional antenna size in PSII. Representative traces of the fluorescence kinetics of DCMU‐treated cells in the presence of 80 (A) or 150 (B) μmol of photons m^−2^ s^−1^ of actinic light at 630 nm. LL, ML, and HL cells are represented in black, red, and blue, respectively.
**Figure S2**. Oxygen evolution of acclimated cells. Oxygen evolution activity of acclimated cells exposed to increasing light intensity. Measurements were normalized with the amount of chlorophyll. LL, ML, and HL cells are represented in black, red, and blue, respectively. Data are reported as the average of three biological replica ± sd.
**Figure S3**. NPQ measurements of acclimated cells. (A) NPQ of LL, ML, and HL cells exposed to increasing light intensity with far‐red off. (B) The same measurements were taken with nigericin‐treated cells. For both the pictures, LL, ML, and HL cells are represented in black, red, and blue, respectively. Data are reported as the average of three biological replica ± sd.
**Figure S4**. Fluorescence traces of *D. giordanoi* cells. The figure reports the fluorescence traces of acclimated cells exposed to a series of saturating pulses with actinic light off (far‐red off). LL, ML, and HL cells are represented in black, red, and blue, respectively.

## Data Availability

The data that support the findings of this study are available from the corresponding author.
