# Supplementary material for: Acclimation of photosynthetic apparatus in the mesophilic red alga Dixoniella giordanoi
Source: Physiol Plant. 2021 Jul 5;173(3):805–17. doi: 10.1111/ppl.13489 (PMC8596783; doi:10.1111/ppl.13489)
Supplement: Supplementary file 1 — Figure S1 Modulation of the functional antenna size in PSII. Representative traces of the fluorescence kinetics of DCMU‐treated cells in the presence of 80 (A) or 150 (B) μmol of photons m−2 s−1 of actinic light at 630 nm. LL, ML, and HL cells are represented in black, red, and blue, respectively. Figure S2. Oxygen evolution of acclimated cells. Oxygen evolution activity of acclimated cells exposed to increasing light intensity. Measurements were normalized with the amount of chlorophyll. LL, ML, and HL cells are represented in black, red, and blue, respectively. Data are reported as the average of three biological replica ± sd. Figure S3. NPQ measurements of acclimated cells. (A) NPQ of LL, ML, and HL cells exposed to increasing light intensity with far‐red off. (B) The same measurements were taken with nigericin‐treated cells. For both the pictures, LL, ML, and HL cells are represented in black, red, and blue, respectively. Data are reported as the average of three biological replica ± sd. Figure S4. Fluorescence traces of D. giordanoi cells. The figure reports the fluorescence traces of acclimated cells exposed to a series of saturating pulses with actinic light off (far‐red off). LL, ML, and HL cells are represented in black, red, and blue, respectively. [file PPL-173-805-s001.pdf]

SUPPORTING INFORMATION

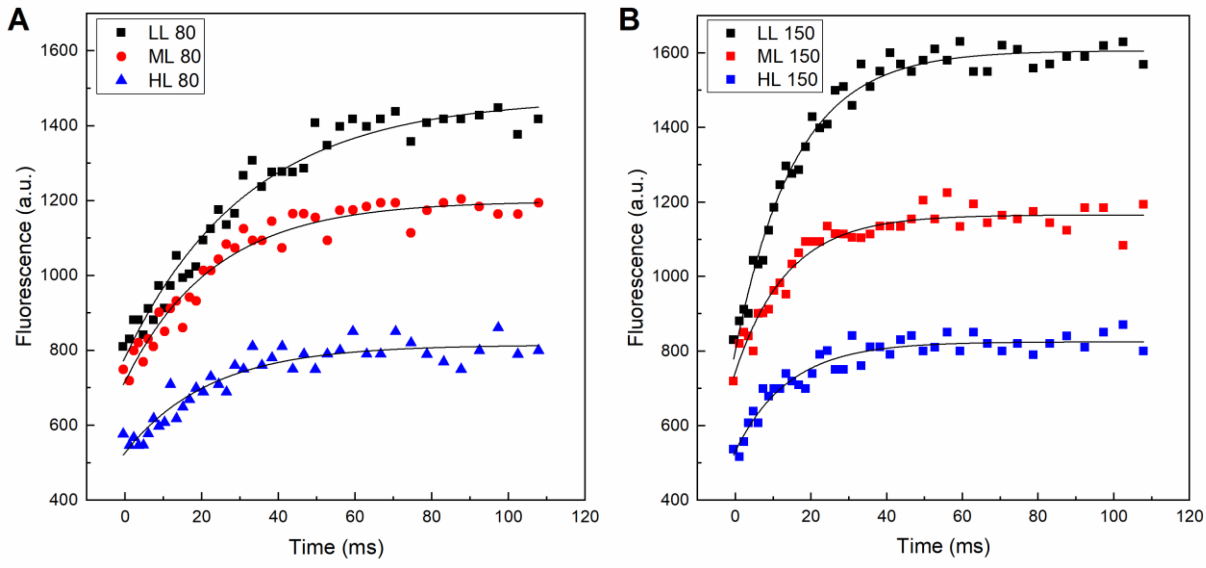

Figure S1. Modulation of the functional antenna size in PSII. Representative traces of the fluorescence kinetics of DCMU-treated cells in the presence of 80 (A) or 150 (B) µmol of photons m^{-2} s^{-1} of actinic light at 630 nm. LL, ML and HL cells are represented in black, red and blue, respectively.

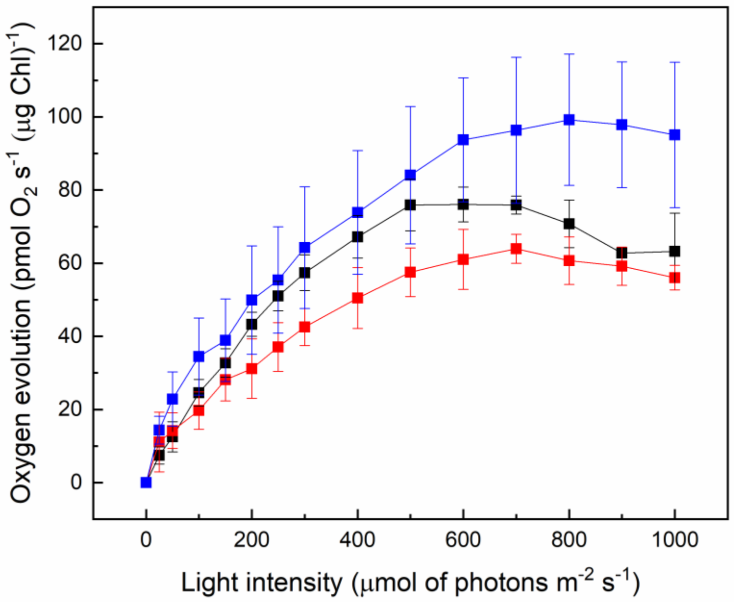

Figure S2. Oxygen evolution of acclimated cells. Oxygen evolution activity of acclimated cells exposed to increasing light intensity. Measurements were normalized with the amount of chlorophyll. LL, ML and HL cells are represented in black, red and blue, respectively. Data are reported as the average of three biological replica ± SD.

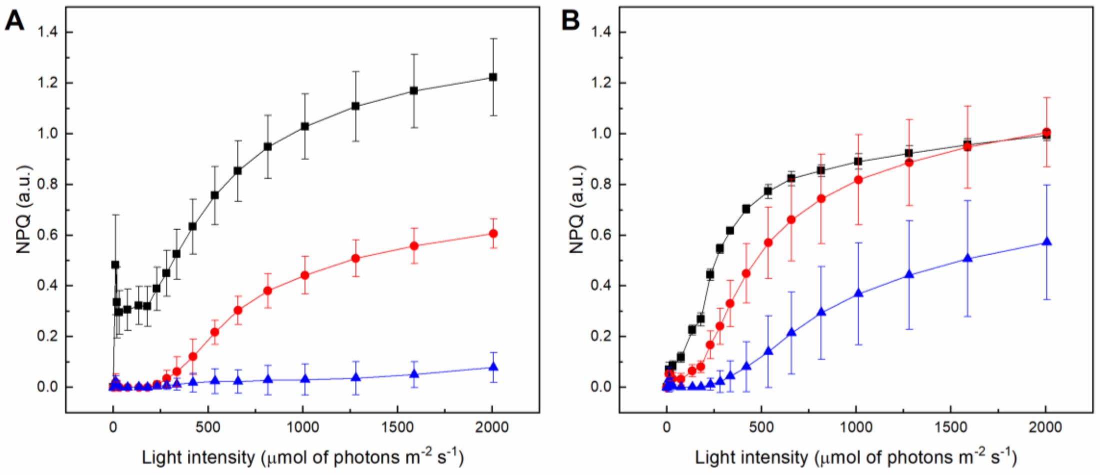

Figure S3. NPQ measurements of acclimated cells. A) NPQ of LL, ML and HL cells exposed to increasing light intensity with far-red off. B) The same measurements were taken with nigericin-treated cells. For both the pictures, LL, ML and HL cells are represented in black, red and blue, respectively. Data are reported as the average of three biological replica ± SD.

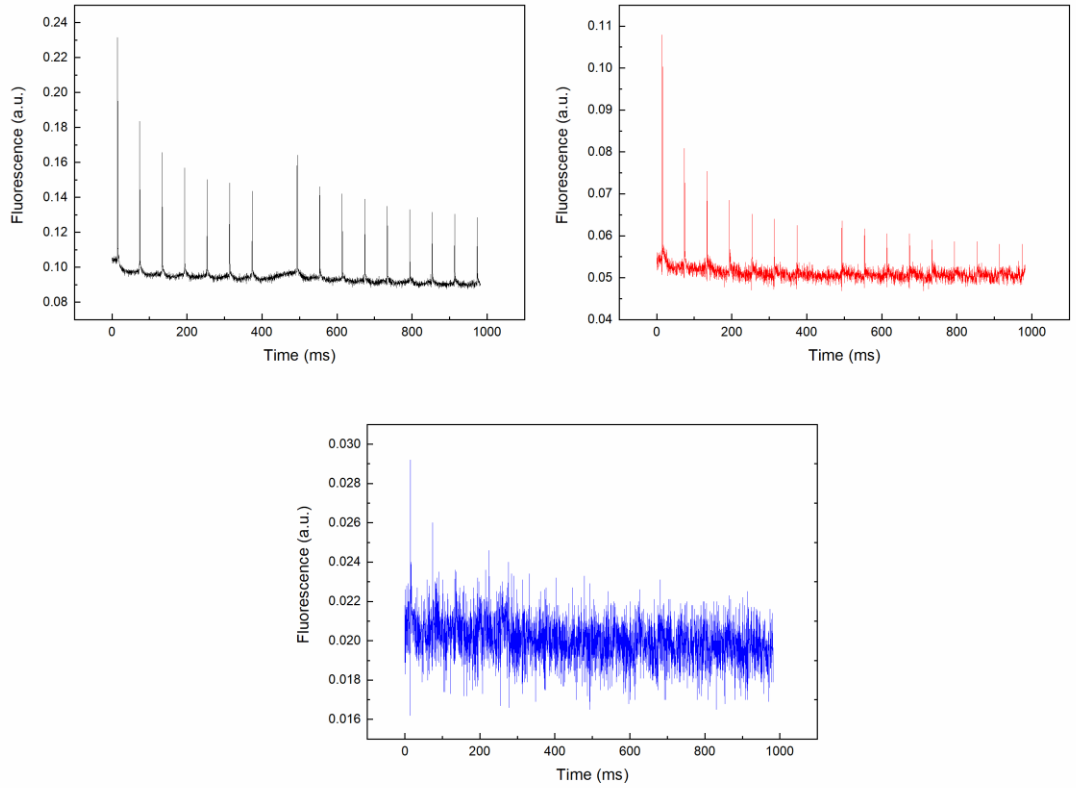

Figure S4. Fluorescence traces of *D. giordanoi* cells. The figure reports the fluorescence traces of acclimated cells exposed to a series of saturating pulses with actinic light off (far-red off). LL, ML and HL cells are represented in black, red and blue, respectively.
